# The association between C-reactive protein and coronary artery calcification: a systematic review and meta-analysis

**DOI:** 10.1186/s12872-024-03856-5

**Published:** 2024-04-10

**Authors:** Amirhossein Tajani, Masoumeh Sadeghi, Navid Omidkhoda, Amir Hooshang Mohammadpour, Sara Samadi, Vahid Jomehzadeh

**Affiliations:** 1 Department of Clinical Pharmacy, School of Pharmacy, Pharmaceutical Research Center, Mashhad University of Medical Sciences, Mashhad, Iran; 2 Metabolic Syndrome Research Center, Mashhad University of Medical Sciences, Mashhad, Iran; 3 Pharmaceutical Research Center, Pharmaceutical Technology Institute, Mashhad University of Medical Sciences, Mashhad, Iran; 4 Department of Internal Medicine, Faculty of Medicine, Mashhad University of Medical Sciences, Mashhad, Iran; 5 Department of Surgery, Faculty of Medicine, Mashhad University of Medical Sciences, Mashhad, Iran

**Keywords:** High sensitivity C-reactive protein, CRP, hs-CRP, CAC, Calcium score, Meta-analysis

## Abstract

**Background:**

While coronary artery calcification (CAC) is recognized as a reliable marker for coronary atherosclerosis, the relationship between the concentration of C-reactive protein (CRP) and the incidence and progression of CAC remains controversial.

**Method:**

PubMed, Embase, Web of Science, and Scopus were systematically searched to identify relevant observational studies until October 2023. The methodological quality of the included studies was evaluated using the Newcastle-Ottawa Scale (NOS). A random-effects meta-analysis was employed to calculate pooled odd ratios (OR) and corresponding 95% confidence intervals, considering heterogeneity among the studies.

**Results:**

Out of the 2545 records, 42 cross-sectional and 9 cohort studies were included in the systematic review. The meta-analysis on 12 eligible cross-sectional studies revealed no significant association between CAC and CRP [pooled OR: 1.03 (1.00, 1.06)]. Additionally, an insignificant association was found between CAC and CRP through meta-analysis on three eligible cohort studies [pooled OR: 1.05 (0.95, 1.15)] with no considerable heterogeneity across studies. Sensitivity analyses indicated that the meta-analysis models were robust. There was no evidence of publication bias.

**Conclusion:**

Based on the meta-analysis findings, elevated levels of CRP did not emerge as a valuable prognostic maker for CAC incidence and progression prediction.

**Supplementary Information:**

The online version contains supplementary material available at 10.1186/s12872-024-03856-5.

## Introduction

Approximately 19 million fatalities worldwide in 2020 were linked to cardiovascular disease (CVD), representing a rise of 18.7% compared to the numbers recorded in 2010 [1]. As a result, preventing CVD is crucial for maintaining public health, as CVD substantially affects nations with high medical costs and economic burdens [2, 3]. Atherosclerosis is caused by lipid buildup in large arteries. Inflammatory pathways are activated as a result of endothelial dysfunction, leading to plaque enlargement, necrotic core formation, and plaque calcification [4] The dysfunctional endothelial cells promote lipid infiltration and leukocyte adhesion, further exacerbating the inflammation and contributing to the progression of the condition [5–9]. CRP is an inflammation-associated protein [10] and is primarily synthesized in hepatocytes. It exhibits a brief lifespan of approximately 18–20 h [11, 12]. In non-inflammatory states, CRP release rate is slow but secreted rapidly after an increase in inflammatory cytokines level, most notably Interleukin 6 (IL-6), Interleukin 1 (IL-1), and Tumor necrosis factor- α (TNF-α) [13, 14]. The American Heart Association has recommended the inclusion of high-sensitivity C-reactive protein (hs-CRP) in the overall assessment of cardiovascular risk [15]. Over time, this concept has evolved, and in 2010, the American College of Cardiology Foundation advised that evaluating CRP levels is a sensible approach for individuals at intermediate risk of cardiovascular events [16]. Coronary artery calcification (CAC) is an advanced feature and indicator of atherosclerosis [17]. It performs better than any cardiovascular risk factor and adds predictive value to traditional equations [18]. CAC can happen via the active or passive route; inflammation is critical for both courses [19]. CAC score is usually calculated by Agatston’s method, and it has been widely used to evaluate the risk of an acute coronary event [20, 21] It is generally known that inflammation causes atherosclerotic plaque instability and promotes the expression of osteogenic factors, which leads to the differentiation of local cells into osteoblast-like cells and calcification. Subsequently, anti-inflammatory factors reduce the expression of osteogenic factors [22–24]. Although inflammation and calcification may seem to work together, the truth is more complicated. Lee H et al. showed that hs-CRP is significantly associated with CAC progression among clinical parameters. Still, the association disappears after adjusting for traditional risk factors [3]. Oh et al. discovered a significant difference in hs-CRP levels among high-risk subjects with CAC scores > 300 [25]. On the other hand, Zeb et al. reported an insignificant association between hs-CRP concentrations and the CAC progression [26]. This study intends to explore the clinical evidence concerning the prognostic significance of CRP as an inflammatory index in assessing the likelihood of CAC development.

## Methods

### Registration and protocol

The present systematic review and meta-analysis followed the meta-analysis of observational studies in epidemiology (MOOSE) guidelines (Supplementary Appendix 1). The protocol was registered in the International Prospective Register of Systematic Reviews PROSPERO (CRD42021242397).

### Databases and search strategy

Until October 2023, four databases, including Pubmed, Scopus, Embase, and Web of Science, were systematically explored to find relevant studies. The population, exposure, comparison, and outcome (PECO) protocol was followed, with participants with symptomatic or asymptomatic CVD as the target population. CRP as the exposure, and CAC as the primary or secondary outcome. Our search was conducted with the MeSH terms of Coronary Artery Calcification, C-Reactive Protein, Coronary Artery Disease, Acute Coronary Syndrome, Ischemic heart disease, ST-elevation Myocardial Infraction, Non-ST Elevation myocardial infraction, Stable Angina, Non-Stable Angina, and Myocardial Infraction. The retrieved studies’ reference lists were examined to find any missing pieces.

### Eligibility criteria and study selection

The two investigators assessed the titles and abstracts of the retrieved records before searching the full text of documents for those intended to fulfill our inclusion criteria. In addition, we requested papers by email twice where the full text was not available, and in case we did not receive an answer, the article was excluded. A third author was enlisted to create certain conclusions to settle disagreements. We included observational studies, including case-control, cohort, and cross-sectional studies, without considering language or publication date. Human research was assessed to determine the relationship between CRP and CAC scores in patients with CVD. The following were excluded: technical reports, conference papers, case reports, animal research, and review articles. Studies involving participants with diseases other than CVD were also excluded.

### Data abstraction and quality assessment

Two reviewers extracted data independently and sorted by first author, year, country, age, population, sample size, effect size (OR, RR, HR) with confidence intervals (CI), study outcome, and results. NOS was used to assess the study’s methodological quality (Supplementary Appendix 2) [27]. A maximum of 9 points were awarded based on selection (4 points), comparability (2 points), and outcome/exposure (3 points) assessment. Scores under 4 indicate low quality, 4–6 moderate, and more than 6 indicate good study quality. All included studies were assessed by two investigators and verified by a third member. Discrepancies in score allocation were resolved by consensus.

### Statistical analysis

We examined the relationship between CRP and CAC using odds ratio (OR) to estimate the effect size. We conducted a random-effects meta-analysis employing the Der-Simonian and Laird method to calculate the combined OR and 95% CIs. Using a random-effects meta-analysis allowed us to consider conceptual and clinical heterogeneity among the studies. We used a forest plot to illustrate the ORs and their respective 95% confidence intervals. To assess the heterogeneity across the studies, we used the I^2^ statistics, where an I^2^ value of 50% or higher suggests significant heterogeneity. Additionally, we employed Cochran’s Q statistic with a significance threshold of *P* < 0.10 to indicate heterogeneity among the studies.

To test the consistency of the results and robustness of the pooled estimates, we conducted sensitivity analysis systematically, removing specific studies or groups of studies (Supplementary Appendix 3). Furthermore, we performed a subgroup meta-analysis to examine the relationship between CAC and CRP based on how CRP was measured, whether in milligrams per deciliter (mg/dl) or Logarithm of milligrams per Liter (log mg/L) and gender. To assess the publication bias, we visually examined funnel plots where we plotted log odds ratios (log ORs) against their standard errors to measure study precision. Also, we conducted Egger’s regression asymmetry test and Begg’s adjusted rank correlation test to evaluate publication bias statistically. We used two-tailed statistical tests, and the significance level was considered less than 0.10. All statistical analyses were conducted using Stata version 14 software developed by STATA Corp. in College Station, TX, USA.

## Results

### Results of the literature search

**Fig. 1:**
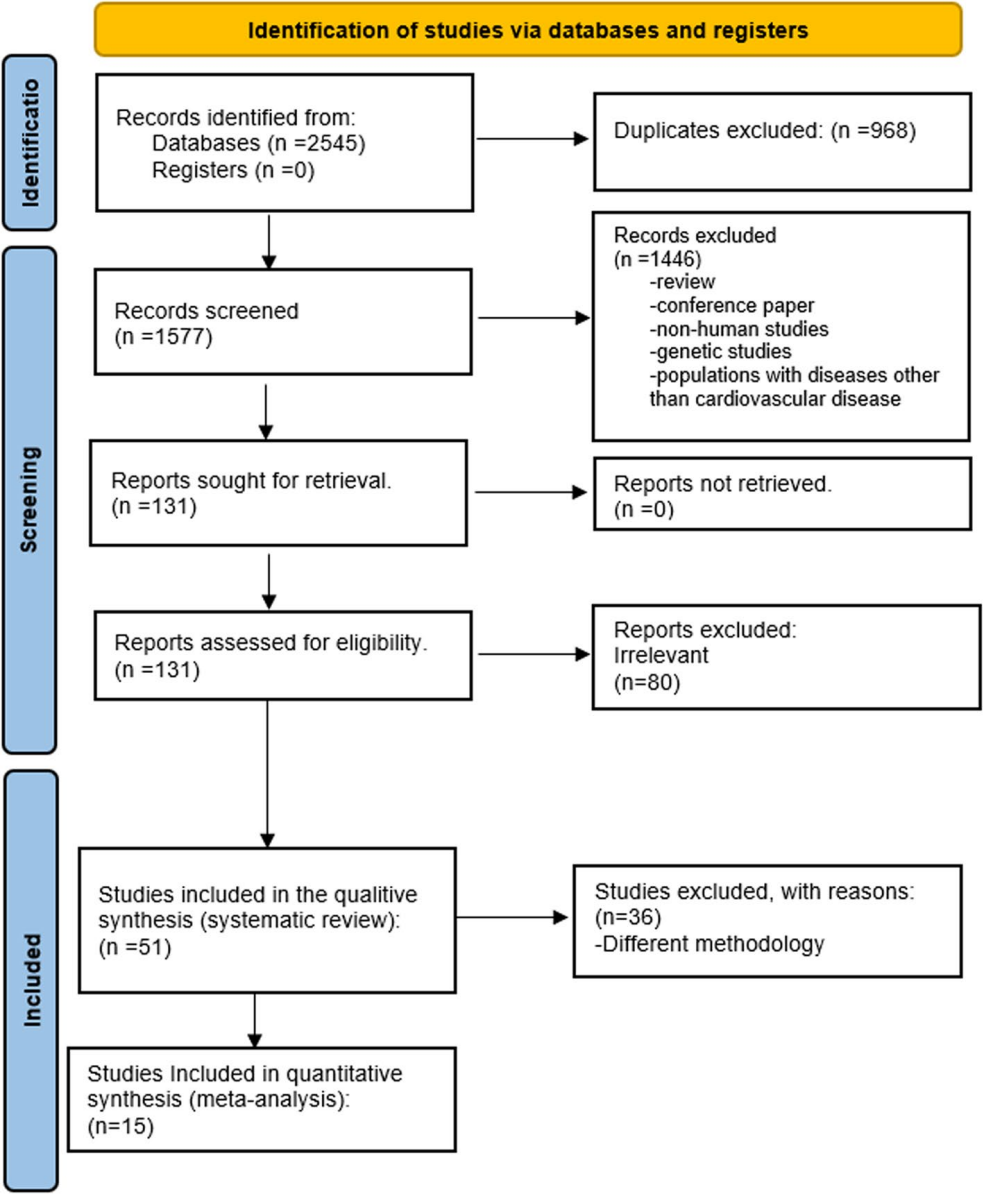
Study selection flow chart

Initially, our systematic review yielded 2545 articles. Among these, 968 were duplicates. After screening the 1577 titles and abstracts, 1446 were excluded, leaving 131 records for full-text examination. Among these 131 articles, 86 reports were discarded as they were reviews, conference reports, or irrelevant to our study; we also collected six articles from looking into references of other papers slightly similar to our work. Finally, 51 studies were chosen for data extraction when they met our inclusion and exclusion criteria. There were 42 cross-sectional studes and 9 cohort studies among the 51 included studes (Fig. 1).

### Descriptive characteristics of the studies

Four cohort studies were conducted in the USA; two used the Multi-Ethnic Study of Atherosclerosis (MESA) database for their research, one used the Early Identification of Atherosclerosis by Noninvasive Imaging Research (EISNER) data, and one utilized Prospective Army Coronary Calcium (PACC) participants. Two studies were done in Asia and three in Europe. Combined, 13,305 patients were studied. The study also included 42 cross-sectional studies with a total of 124,606 participants. Thirty-six research involved both genders; five included only men [28–32], and one recruited just women [33]. This analysis comprised 42 cross-sectional studies, 14 of which were conducted in the United States, four in South America and Brazil, three in Europe, and 21 in Asia. The data sources of population-based cross-sectional studies included participants from MESA, the Brazilian Longitudinal Study of Adult Health, the ERA-JUMP study, the Study of Inherited Risk of Coronary Atherosclerosis (SIRCA), the Genetic Network of Arteriopathy (GENOA) study, the Framingham Heart Study (FHS), the Dallas Heart Study (DHS), the Shiga Epidemiological Study of Subclinical Atherosclerosis (SESSA), the Mediators of Atherosclerosis in South Asians Living in America study (MASALA), The Swedish CArdioPulmonary bioimage Study (SCAPIS), and the Study of Women’s Health Across the Nation (SWAN).

### Association between CRP and CAC in cohort studies

#### Systematic review findings

Characteristics of cohort studies are presented in Table 1. In a longitudinal study, Zeb et al. investigated the association of inflammatory markers and lipoprotein particle subclasses with the incidence and progression of CAC. Their findings showed no association between hs-CRP and CAC incidents. [RR = 0.97 [0.92, 1.02] *p* < 0.001] [26]. In 2021, research on subjects with CVD risk factors or atypical chest pain revealed a significant association between hs-CRP ≥ 2.0 mg/L and CAC progression, HR:1.18 (1.01–1.63), but only in the univariate analysis [3]. Intriguingly, Nankeolyar et al. found an inverse association between hs-CRP and CAC measured at baseline but failed to produce an association with CAC when measured continuously or categorically [34]. Diederichsen et al. explored the association between 15 biomarkers and CAC in a population of 1006 particiants and followed up with a second computed tomography scan after five years but couldn’t find an independent association between CAC growth and CRP [35]. In a study by Gauss et al., the association of inflammatory markers with CAC and epicardial fat volume was investigated. Consistent with previous studies, they exhibit no association between hs-CRP and baseline levels or progression of CAC [36]. A study performed on the MESA cohort with low Framingham risk scores pursued whether novel markers that don’t require ionizing radiation can forecast the progression of CAC. In univariate and age-adjusted models, CRP was associated with only increased CAC, not incident CAC. However, this association became non-significant in multivariable models adjusted for age and other traditional CVD risk factors in low-risk participants [37]. In an investigation by Hamer et al. on 466 healthy men and women from the Whitehall II epidemiological cohort, no significant association was found between CAC and CRP after adjusting for follow-up time, pre-stress cortisol level, employment grade, statin use, resting blood pressure, fibrinogen, high-density lipoprotein cholesterol (HDL-C) and low-density lipoprotein cholesterol (LDL-C), body mass index (BMI), and smoking [38]. Lastly, Taylor et al. reported that CRP levels were similar in univariate analysis in those with and without CAC progression [39].

**Table 1:** Characteristics of Cohort studies

Study ID	Population	Place/Source	Age(years)	Sample Size/Follow-up	OR/HR/RR (CI95%)	Results	NOS
[3]	cardiovascular risk factors or atypical chest pain	South Korea	56.4 ± 7.2	1015 / 39 month	^a^HR:1.28 (1.04, 1.67)	Association in Univariate Analysis	Good
[26]	free of clinical cardiovascular disease	USA/MESA	61.8	6067/ 6.5 ± 3.5 years	RR: 0.97 (0.92, 1.02)	No association	Good
[40]	baseline zero CAC score	Taiwan	51.17 ± 8.24	830/4.55 ± 2.42 years	OR: 1.399 (0.923, 2.123)	No association	Good
[34]	Asymptomatic intermediate ASCVD risk factors	USA/EISNER	62.5 ± 8.3	1207/4 years	OR: 0.95	No association	Good
[35]	random sample from the general population	Denmark	55.39 ± 5.01	1006/ 5 years	IRR: 1.01 (0.96, 1.06)	No association	Good
[36]	with an enhanced risk for CAD	Germany	58.8 ± 10.0	94/1.9 ± 0.5 years	N/A	No association	Good
[37]	classified as low risk for CHD events	USA/MESA	60.4 ± 9.2	2620/2.5 years	OR: 1.07 (0.97, 1.18)	No association	Good
[38]	no history of signs of CHD, hypertension, inflammatory diseases, or allergies	United Kingdom	63.36 ± 5.9	466/ 3 years	OR: 0.99 (0.89, 1.10)	No association	Good
[39]	healthy asymptomatic	USA/PACC	47.9 ± 2.8	180/ 2 years	OR: 1.23 (0.73, 2.08)	No association	Good

*OR *Odds Ratio, *HR *Hazard Ratio, *N/A *Not announced

^a^Unadjusted

#### Meta-analysis findings

**Fig. 2:**
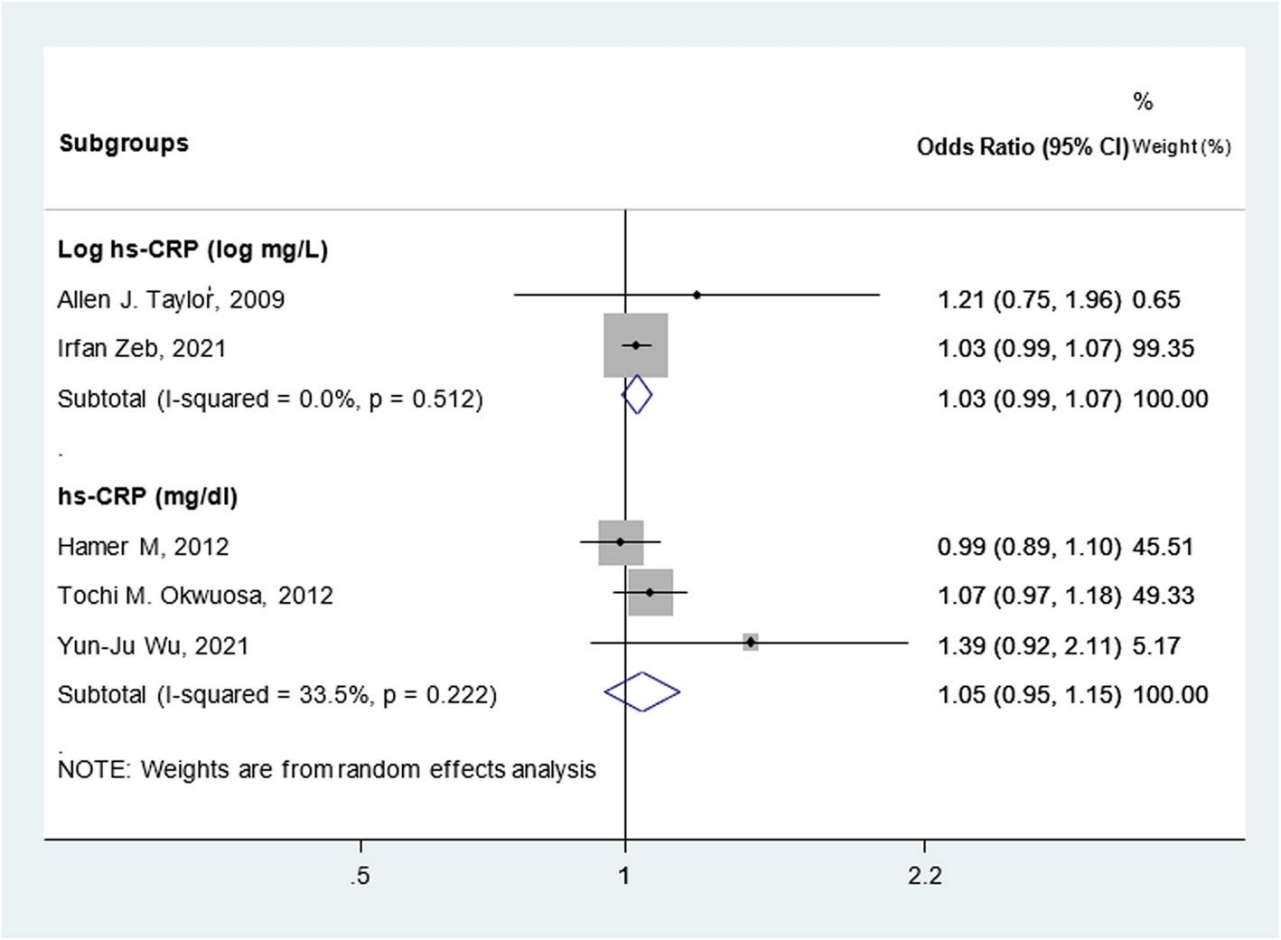
Meta-analysis of cohort studies investigating the association between CRP and CAC separated by CRP measurement scale. Diamond represents the summary odds ratio (pooled OR) estimate, and its width shows a corresponding 95% CI with random effects estimate. The square’s size and central point reflect the study-specific statistical weight (inverse of the variance), and the point estimate of the OR and horizontal line reflects the corresponding 95% CI of the study. I^2^ test and Cochran’s Q statistic were used to assess the statistical heterogeneity (*P* < 0.10) across studies

Seven cohort studies were primarily excluded from the meta-analysis because their data could not be included in the quantitative synthesis. These studies employed various statistical methodologies to report the relationship between CRP and CAC, and they analyzed different variables in their studies. The meta-analysis on three cohorts showed no significant association between CAC and hs-CRP [OR: 1.05(0.95, 1.15)] (Fig. 2). We also presented the results of two studies that measured hs-CRP concentration on a Log mg/L scale [OR: 1.03(0.99, 1.07)]. No considerable heterogeneity was found across studies. Sensitivity analysis demonstrated the consistency of the results, indicating that the meta-analysis model was robust.

### Association between CRP and CAC in cross-sectional studies

#### Systematic review findings

Out of 42 included cross-sectional studies in our paper, five studies [25, 41–44] reported a positive association between CAC and CRP levels. Another five studies [45–49] also reported a positive association but only in particular populations, for example, in very elderly or diabetic women or women of African-American origins. The remaining thirty-one studies declared no association between CAC and CRP levels (Table 2).

**Table 2:** Characteristics of cross-sectional studies

Study ID	Population	Location/Source	Age	Sample Size	Effect Size	Results	NOS
[50]	Asymptomatic randomly selected	Sweden/SCAPIS	50–64	25,408	OR:0.96(0.88, 1.04)	No association	Good
[2]	Asymptomatic above 40 years	South Korea	55.9 ± 7.7	617	OR:1.202 (0.830, 1.741)	No association	Good
[51]	patients with chest pain	Turkey	57.5 ± 8.6	187	N/A	No association	Good
[28]	Centrally Obese Non-Diabetic Men	Indonesia	55.20 ± 6.51	60men	Correlation Coefficient (r): 0.077	No association	Good
[52]	free of clinical ASCVD	USA/MESA	61 ± 10	5196	N/A	No association	Good
[29]	aged 40–79 randomly selected men	Japan/SESSA	69.5 ± 6.9	1035 men	OR:1.13(0.97, 1.32)	No association	Good
[53]	civil servants aged 35–74 years	Brazil/ELSA	58	3753	OR:1.28(0.96, 1.70)	No association	Good
[41]	candidates for computed tomography	Iran	60 ± 10.5	200	OR:11.8 (3.39,41.29)	Positive association	Weak
[54]	asymptomatic immigrant	USA/MASALA	55 ± 9	906	OR:1.17 (0.74, 1.85)	No association	Good
[55]	Stable without known CAD	Brazil	63.2 ± 9.6	130	OR: 1.25 (0.34, 4.63)	Not Associated	Good
[56]	chest pain ± positive treadmill test ± presence of multiple coronary risk factors	54.83 ± 11.63	379	OR:1.090(1.007, 1.180)	No association	Good	
[57]	Tsimane villagers, age < 40	USA	40–60	705	N/A	No association	Good
[42]	people without known CAD	China	59.2 ± 14.92	348	N/A	Positive association	Good
[58]	individuals who underwent a non-contrast CT scan and echocardiography	South Korea/CAESAR	51.1 ± 9.3	2276	N/A	No association	Good
[33]	age 42 to 52 years, an intact uterus, menstruating within the prior three months	USA/SWAN	51.3 ± 2.8	372 women	OR:1.16 (0.88, 1.53)	No association	Good
[59]	Healthy Individuals	USA/MESA	70 ± 8	6745	N/A	No association	Good
[60]	underwent helical computed tomography	China	67 ± 11	125	N/A	No association	Good
[25]	men aged ≥ 45 women aged ≥ 55	South Korea	66 ± 6	456	OR:2.812 (1.600, 4.942)	Positive association	Intermediate
[61]	patients who underwent coronary CT	China	68.38 ± 10.05	157	N/A	No association	Good
[30]	selected randomly for enrollment men	Japan/ERA-JUMP	40–49	904 men	OR:0.96(0.80, 1.15)	No association	Good
[62]	randomly sampled from national registries	Denmark/DanRisk	50–60	1126	OR:1.00 (0.98, 1.03)	No association	Good
[46]	the absence of atherosclerotic coronary, cerebrovascular, or peripheral artery disease	Brazil	85 ± 4	208	OR:3.05 (1.25, 7.42)	Association in Special Population	Good
[63]	Presence of chest pain/ Abnormal Stress test/ Asymptomatic with multiple ASCVD	USA	57 ± 1.0	92	N/A	No association	Good
[45]	participated in a comprehensive health examination in 2010	South Korea	42.20 ± 6.89	12,030	N/A	Associated when grouped with HDL	Good
[43]	randomly a selected group of MESA participants	MESA/USA	66.96 ± 9.2	1827	N/A	Positive association	Good
[64]	employees and family members of various industrial companies	Korea	41.36 ± 7.0	24,063	OR:0.932 (0.743, 1.169)	No association	Good
[31]	Male subjects from Samsung Medical Center	South Korea	58% above 50 years	3408men	N/A	No association	Good
[32]	Male participants from Samsung Medical Center	South Korea	57% above 50 Years	3010 men	N/A	No association	Good
[65]	participants with clinical suspicion of CHD	Japan	68.0 ± 11.0	1363	N/A	No association	Good
[66]	low-intermediate risk for CAD	Turkey	53 ± 10	272	N/A	No association	Good
[67]	Asymptomatic individuals	South Korea	30–86	531	N/A	No association	Good
[68]	Healthy Individuals	Germany	61.3 ± 8.7	455	N/A	No association	Good
[49]	consecutive patients aged 80 years or over Asymptomatic	Brazil	85 ± 5	184	N/A	Association in Special Population	Good
[48]	Healthy Individuals	USA/MESA	64.7 ± 8.7	3046	OR:1.12 (0.96, 1.31)	No association	Good
[47]	type 2 diabetes and without clinical evidence of CVD	USA/PDHS-SIRCA	35–75	2159	N/A	Association in Special Population	Good
[69]	Healthy Individuals	USA/MESA	45–84	6783	RR: 1.05 (0.99,1.12)	No association	Good
[70]	asymptomatic, ethnically Middle Eastern Egyptians	Egypt	50.6 ± 5.9	177	OR:1.101(0.846, 1.431)	No association	Good
[71]	DHS Participants	Japan/USA	46.3±9.4	3373	OR:1.02 (1.0, 1.04)	No association	Good
[72]	patients with symptoms of typical or atypical chest pain	Taiwan	73±1	124	N/A	No association	Good
[73]	Hispanic white hypertensive subjects	USA	66±7	354	N/A	No association	Good
[74]	Asymptomatic subjects with a family history of premature CAD	USA/SIRCA	Men: 30–65 Women: 35–70	914	OR: Men: 0.92(0.83, 1.04) Women: 1.02(0.94, 1.08)	No association	Good
[44]	Healthy Individuals	USA/FHS	5–70	321	N/A	Positive association	Good

*N/A *Not announced, *IRR *Incident Rate Ratio, *RR *Relative Risk

A population-based cross-sectional study recruiting 3753 participants revealed no increase in the levels of hs-CRP or GlycA as examined in higher CAC cut-points [53]. An investigation using 1827 individuals with a mean age of 66.96 ± 9.2 from the MESA cohort study demonstrated that in a fully adjusted model, including lipid and glucose measures, the connection between CRP, IL-6, and fibrinogen with CAC persisted as statistically significant. To elaborate, an increase of one standard deviation (SD) in CRP was linked to around a 5% higher prevalence of CAC (95% CI: 1.03, 1.08) [43]. As reported by Okwuosa et al., no statistical association was indicated between CRP levels and CAC [37]. Arps et al., in a study on MESA participants, found a simple association between higher CAC scores and co-morbid atherosclerotic cardiovascular disease (ASCVD) risk factors such as old age, male sex, and white race. Still, CRP was not one of these risk factors [52]. Using 6745 participants of MESA, Kummuri et al. exhibited a strong positive correlation with an increase in CAC score in all anthropometric measurements, except for BMI. However, hs-CRP did not show a significant distribution pattern in this context [59]. Sorensen et al. reported that the likelihood of falling into a higher category of CAC was not associated with increased levels of hs-CRP [62]. Sung et al., in the multivariable fully-adjusted regression analysis, nitoced that individuals with high CRP concentrations and low-level HDL-C are more prone to have CAC score > 0 compared to a group with low levels of CRP and high-level HDL-C [45]. Furthermore, Rhee et al. found no correlation between any groups of increasing CAC categories and hs-CRP [64]. Qasim et al., in a model for diabetic women, adjusted for age, race, medication, metabolic syndrome, Framingham risk score, and BMI, observed a significant association between CAC and CRP. This relationship was attenuated in non-diabetic women, and there was no such association in diabetic or non-diabetic men [47]. Utilizing MESA participants, although in the age, sex, and ethnicity-adjusted model, an increased risk of 13% was reported in those in the highest quartile of CRP for CAC, adding traditional coronary heart disease risk factors demonstrated insignificant association between CAC and CRP [69]. Khera et al., in a multivariable analysis adjusted for traditional CVD risk factors, estrogen, and statin medication use, revealed no significant association between CRP levels and CAC [71]. Recently, a study recruiting 25,408 SCAPIS cohort participants from Sweden demonstrated a significant association between high hs-CRP and CAC by univariate analysis; however, after stepwise adjustments, especially after adjusting for BMI, the association attenuated and wasn’t meaningful anymore [50].

#### Meta-analysis findings

**Fig. 3:**
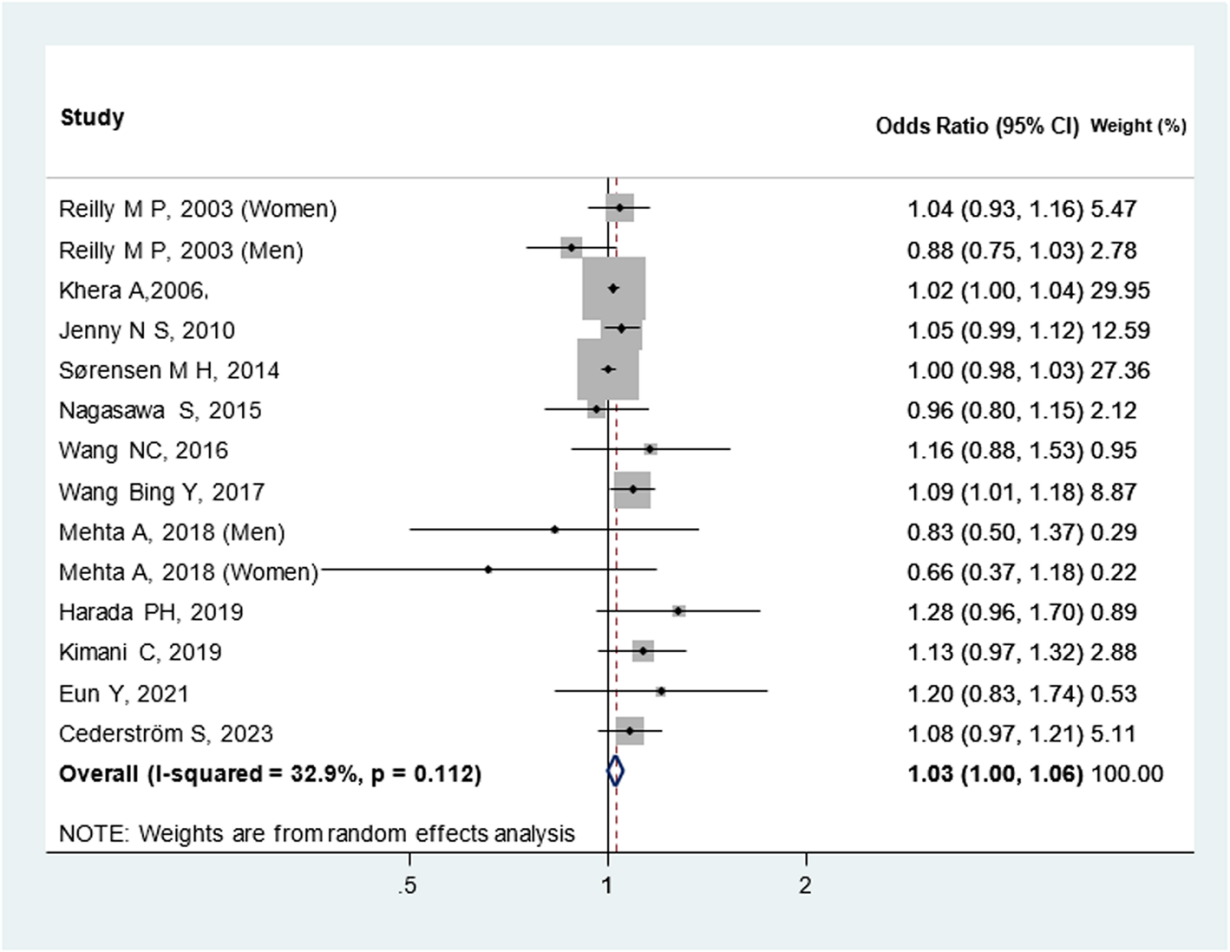
Meta-analysis of cross-sectional studies investigating the association between CRP and CAC. I^2^ test and Cochran’s Q statistic were used to assess the statistical heterogeneity (*P* < 0.10) across studies

Overall, twelve cross-sectional studies were included in the meta-analysis, and all of them showed no statistically significant association between CAC and CRP (Fig. 3) [OR: 1.03 (1.00, 1.06)].

**Fig. 4:**
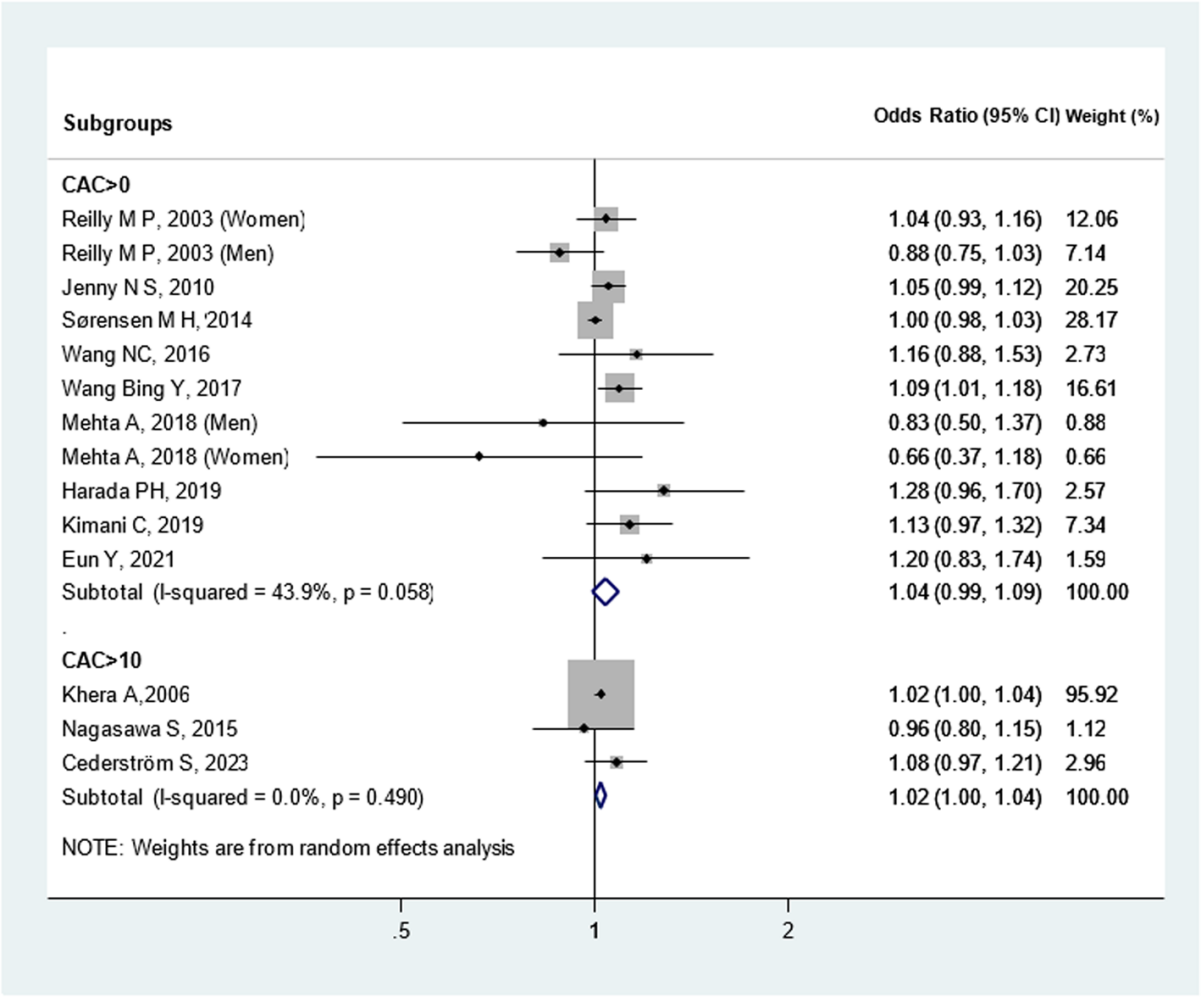
Meta-analysis of cross-sectional studies investigating the association between CRP and CAC (separated by cut of CAC presence)

**Fig. 5:**
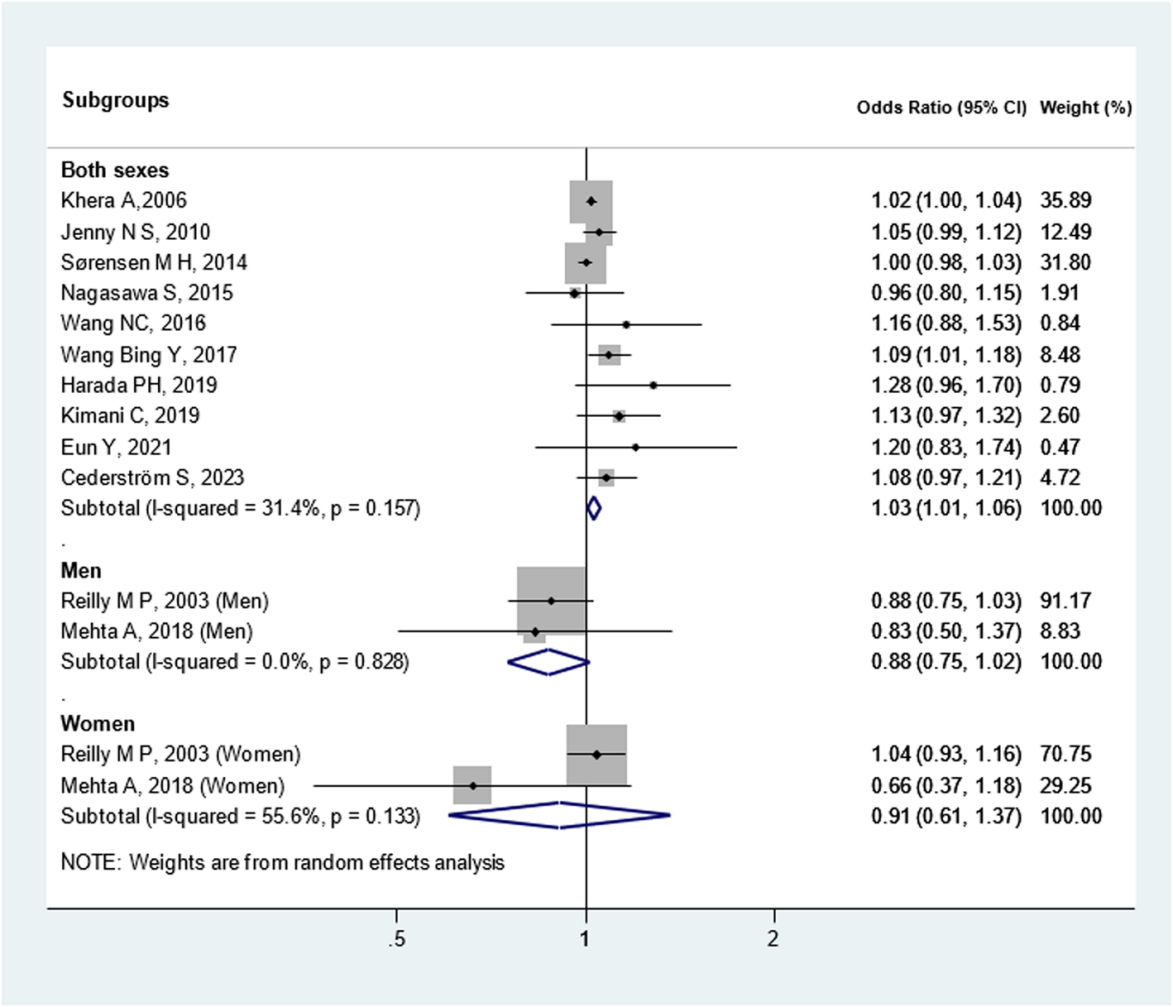
Meta-analysis of cross-sectional studies investigating the association between CRP and CAC (separated by sex)

**Fig. 6:**
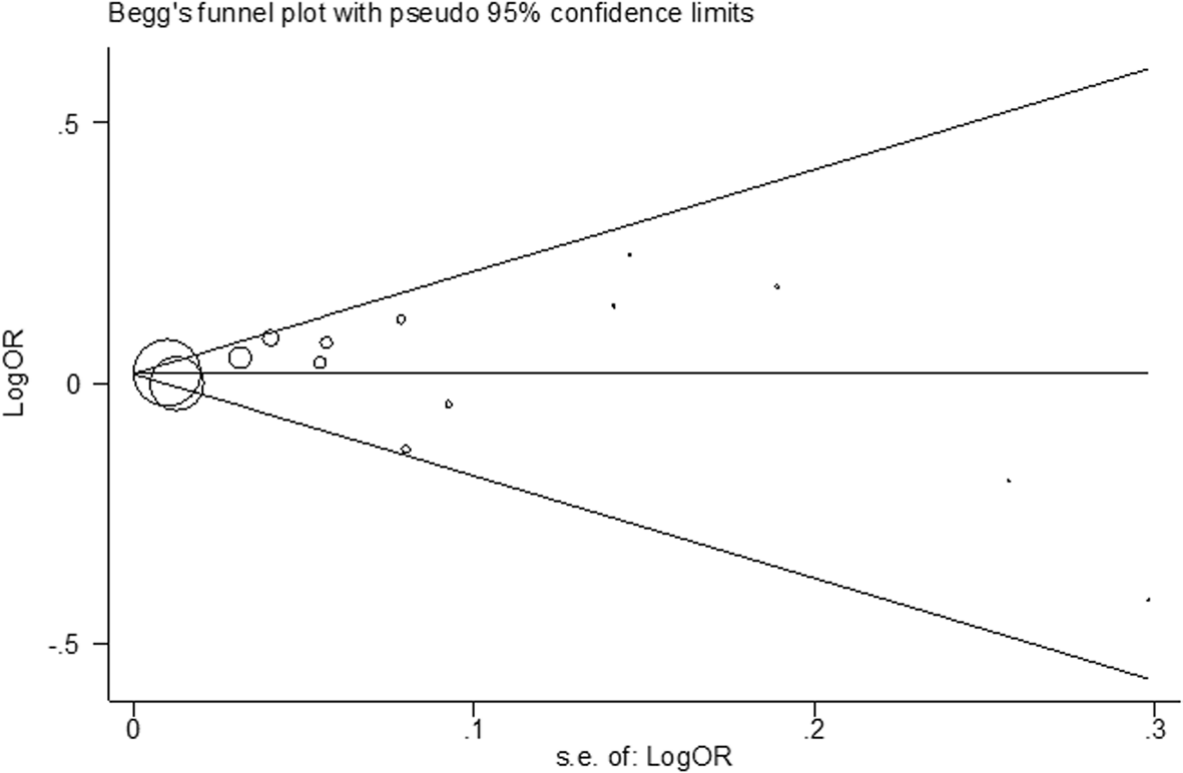
Begg’s funnel plot for assessing the presence of publication bias. The logarithm of the odds ratio was plotted against the precision of the study (*P* = 0.61 for Egger’s regression asymmetry test). There was no evidence of publication bias statistically (*P* = 0.15 for Egger’s regression asymmetry test)

We also evaluated these twelve studies based on CAC cut-offs (Fig. 4) and sex (Fig. 5). When stratified according to CAC thresholds, no notable association of CAC > 0 [OR: 1.04(0.99, 1.09)] and CAC > 10 [OR: 1.02(1.00, 1.04)] with CRP was observed. Additionally, upon analysis separated by gender, elevated CRP levels showed an insignificant relationship between CAC and CRP in men [OR: 0.88(0.75, 1.02)] and women [OR: 0.91(0.61, 1.37)]. These preliminary findings warrant further validation through more rigorous research methodologies and longitudinal cohort studies. Additionally, Heterogeneity among cross-sectional studies was not found. Sensitivity analysis demonstrated consistent results, so the meta-analysis model was robust. There was no evidence of publication bias visually and statistically (P = 0.62 for Begg’s adjusted rank correlation test and P = 0.45 for Egger’s regression asymmetry test) (Fig. 6).

## Discussion

Our systematic review and meta-analysis examined 15 observational studies, including 12 cross-sectional and three cohort studies, to describe the relationship between CRP and CAC. The majority of the studies have stated no association between CRP and CAC. Nevertheless, several studies indicated a statistically significant correlation between CRP and CAC, whether in univariate or multivariable analysis. Moreover, our results showed no significant role of CRP in risk stratification for CAC score, supporting a different pathophysiology approach of CRP and CAC development. Therefore, additional prospective cohort studies must be conducted, including well-designed methodologies and recruiting healthy individuals. To validate the association, these studies should examine the relationship between CRP and CAC at baseline and follow-up stages.

Measuring CAC can be costly and involve radiation exposure. Recent studies showed the potential role of novel biomarkers as a prognostic value in predicting CAC score [75–79], suggesting novel pharmacological targets in reducing CAC burden.

The recent guidelines from the American Heart Association emphasize the importance of performing CAC testing for predicting cardiovascular events and stratifying CVD risk. This underscores the significance of CAC as a diagnostic marker in CVD [80–84].

CRP is a useful marker in assessing inflammation because it rapidly increases in concentration following a stimulus, reaching its peak around two days later, and takes several days to return to baseline levels, indicating the duration and intensity of the inflammatory response [85–90].

Local vascular inflammation is linked to calcification. It’s widely recognized that inflammation within atherosclerotic lesions plays a significant role in triggering the rupture of these plaques [23].

Local factors, including inflammatory cytokines stimulate the transformation of vascular smooth muscle cells (VSMCs) into osteogenic cells. On the other hand, unsaturated fatty acids like eicosapentaenoic acid can counteract this process by reducing the expression of factors related to bone formation [24]. Clinical evidence has established a direct connection between vascular inflammation and the development of calcification. Interestingly, positron emission tomography (PET) reveals that vascular inflammation tends to occur before calcification becomes visible through standard tomography [91]. Furthermore, research findings suggest that inflammation in the vascular system can fluctuate over time, and each episode of acute inflammation might contribute to the progression of calcification. Importantly, from a clinical standpoint, it’s unclear whether calcification can trigger inflammation [19].

Our nine included cohort studies found no significant association between CAC and CRP except for one, Lee H et al. In a retrospective observational cohort study, they investigated 1015 Korean subjects who underwent CAC scoring by computed tomography. During 39 months of follow-up, they found a significant association between CAC progression and CRP in men and not women. However, the association was not adjusted for additional risk factors. Also, male subjects were the predominant study population (80.6%), a big difference from the general composition of society [3].

In thirty-two of our included cross-sectional studies, researchers found no association between CRP and CAC. In the remaining ten studies, however, Oh et al. investigated 456 participants from South Korea; subjects with a CAC score of ≥ 300 agatston units, in univariate regression analysis, found log hs-CRP to be significantly associated with the high CAC group [OR: 2.812 (1.600,4.942)]. Nevertheless, the study with cross-sectional design and no adjustment for conventional CVD risk factors, failed to verify the association between CAC and CRP [25]. In another study, Fu et al. found an association between CAC and hs-CRP in multiple linear regression analysis. However, they only included subjects with complaints of chest pain in a cross-sectional manner, making it challenging to establish a clear causal link [42]. In the research involving individuals without any evident cardiovascular issues, Wang et al. observed a connection between CRP levels and the presence of CAC in both male and female participants. This association remained significant even after considering age, individual conventional risk factors, and Framingham risk score. Nonetheelss, CRP was measured 4 to 8 years before conducting electron beam computed tomography; it’s possible that the relationship between CRP and the presence of CAC was influenced by the progression of atherosclerosis in individuals who had elevated CRP levels [44]. The remaining cross-sectional studies found an association between CAC and CRP, but the association can not be generalized to the community. In this regard, Qasim et al. found a significant link between CAC and CRP levels in type 2 diabetic women. Conversely, there was no evident relationship between CRP and CAC in diabetic or nondiabetic men [47]. Sung et al. found CAC score > 0 to be significantly associated with high CRP concentration levels in individuals with low HDL-C [45]. Additionally, Quagli et al. discovered a distinct and independent relationship between CRP levels and the presence of CAC in older adults (aged 80 years or older) without underlying health issues [46].

Based on the pooled estimation in our study, sub-group analysis by gender and CAC threshold revealed no significant relationship between CAC and CRP. To confidently address this issue, however, more research is needed, specifically focusing on gender-specific analysis.

Research conducted in animal models has yielded mixed findings. In a study by Paul et al., they observed a correlation between the presence of CRP in atherosclerotic plaques and enlargement in their size [92]. On the other hand, Tennent et al., who worked with mice expressing transgenic human CRP, found no indications of heightened atherosclerotic buildup, increased complexity of these lesions, or occurrences of spontaneous thrombosis and plaque-related fatalities [93].

The current systematic review comes with certain limitations. CRP concentrations were measured by traditional and highly sensitive methods, which can detect low levels of chronic inflammation suited for detecting CVD. This might lead to the heterogenicity in results.

The variability in recorded CAC cutoff values has led to heterogenicity in findings among different studies.

The studies exhibited differences in several aspects, including the ratio of males to females, the existence of other cardiovascular risk factors among participants, the presence of documented CVD, the duration of the follow-up period, and the average age of the subjects.

Therefore, conducting additional primary studies, particularly prospective cohort studies with larger sample sizes, while considering the shortcomings of previous research could help confirm the link between CRP and CAC score.

## Conclusion

The present meta-analysis findings indicate no significant association between high CRP levels and CAC in cohort and cross-sectional studies. Therefore, it appears that CRP concentration cannot be used as a predictor to determine the necessity of CAC measurement and screening. Hence, it seems imperative to carry out large-scale prospective cohort studies. These investigations ought to employ robust methodologies and entail recruiting individuals in good health. To validate the observed association, the focus should be on investigating the correlation between CRP and CAC during both the initial assessment and follow-up phases.

### Supplementary Information

**Supplementary Material 1.**

## Data Availability

The datasets generated during and/or analyzed during the current study are available from the corresponding author on reasonable request.
